# Effects of Hypertension, Uric Acid Level, and Other Physiological Factors on Blood Lithium Concentration/Dose Ratio Values in Patients With Manic Episodes

**DOI:** 10.31083/AP47753

**Published:** 2025-10-28

**Authors:** Zijun Xiong, Qin Li, Mingchao Li, Dan Liao, Mingyan Luo, Ying Xia, Fei Xiao, Yulin Cao, Zou Su, Qiuming Ji

**Affiliations:** ^1^Department of Laboratory, Wuhan Wudong Hospital, 430084 Wuhan, Hubei, China; ^2^Department of Psychiatry, Wuhan Wudong Hospital, 430084 Wuhan, Hubei, China; ^3^Department of Psychiatry, Wuhan Mental Health Center, 430012 Wuhan, Hubei, China; ^4^Department of Science, Wuhan Wudong Hospital, 430084 Wuhan, Hubei, China

**Keywords:** manic episode, lithium, hypertension, C/D value, uric acid

## Abstract

**Objective:**

To explore the effects of hypertension, uric acid (UA) level, and other physiological factors on blood lithium concentration/dose ratio (C/D; a measure of lithium pharmacokinetics) values in patients experiencing manic episodes.

**Methods:**

A total of 644 patients with manic episodes were treated at the study hospital between January and June 2022. Patients were divided into groups according to their systolic and diastolic blood pressure (BP) as well as blood glucose, triglyceride, and UA levels. The effects of these factors on blood lithium C/D values after lithium carbonate treatment were examined.

**Results:**

The mean blood lithium C/D value of all study participants was 0.832 ± 0.248 mmol·L^-1^·g^-1^·d. There was no significant difference in blood lithium C/D value between patients with abnormal and normal diastolic BP (*p* > 0.05). However, patients with an abnormal systolic BP (>130 mmHg) had lower lithium C/D values than those with normal systolic BP (*p* < 0.05). Systolic BP was negatively correlated with C/D value (*r* = –0.232; *p* = 0.001), as was UA level (*r* = –0.114; *p* = 0.013).

**Conclusion:**

Hypertension and elevated UA levels can affect the blood lithium C/D value in patients with manic episodes. Personalized treatment approaches that take these physiological factors into account may help reduce treatment risks.

## Main Points

1. Systolic blood pressure (BP) is negatively correlated with lithium concentration/dose ratio (C/D) value in patients with manic episodes.
2. Uric acid (UA) levels are also negatively correlated with lithium C/D value.
3. Patients with abnormal systolic BPs had lower lithium C/D values than those with normal systolic BPs.
4. These findings suggest the need for personalized lithium dosing in patients with hypertension and abnormal UA levels.
5. Regular monitoring of BP and UA level may help to optimize the treatment of manic episodes with lithium.

## 1. Introduction

Manic episodes are periods of abnormally elevated mood and energy that can occur in patients with bipolar disorder and other conditions. These episodes significantly impact patients’ physical and mental health, with severe cases potentially leading to self-harm or suicide. Since the 1950s, lithium carbonate has been widely used as an effective anti-manic and prophylactic medication for mood disorders [1]. International treatment guidelines recommend lithium salts as the first-line therapy in preventing the occurrence of manic episodes [2].

Manic episodes have been associated with abnormal regulation of neurotransmitters, particularly dopamine (DA) [3]. Lithium ions can inhibit the release of neurotransmitters, such as norepinephrine and DA, while also promoting their re-uptake and inactivation in the synaptic cleft. This mechanism helps to treat manic episodes by reducing neurotransmitter concentrations [4].

Patients experiencing manic episodes often present with comorbid conditions such as hypertension, hyperglycemia, and hyperlipidemia, with hypertension being the most prevalent comorbidity [5]. Hypertension can cause changes in renal structure and function, potentially leading to hypertensive kidney injury [6]. Such kidney injuries can reduce the glomerular filtration rate (GFR) [7], which is closely related to the metabolism of lithium in the body [8].

Lithium carbonate has a narrow therapeutic window, with its therapeutic concentration typically within 0.6–1.2 mmol [9]. Insufficient serum lithium concentrations lead to ineffective treatment, while excessive concentrations can cause significant toxicity and side effects. These can range from mild symptoms, such as diarrhea and limb tremors, to severe complications, such as decreased renal function [10] and hypothyroidism [11].

Given the complex interplay between lithium pharmacokinetics and various physiological factors, it is crucial to understand how conditions such as hypertension and other metabolic abnormalities might affect lithium metabolism. The concentration/dose ratio (C/D) of lithium, which represents steady-state serum concentration relative to administered dose, provides valuable information about an individual’s lithium pharmacokinetics.

The purpose of this study is to investigate how hypertension, uric acid (UA) level, and other physiological factors affect blood lithium C/D values in patients with manic episodes. By understanding these relationships, we aim to improve the safety and efficacy of lithium carbonate treatment through more personalized dosing strategies.

## 2. Materials and Methods

### 2.1 Study Participants

A total of 644 patients with manic episodes who were treated with lithium carbonate at the study hospital between January, 2022 and June, 2022 were retrospectively included using convenience sampling. The study was conducted in accordance with the Declaration of Helsinki and the study protocol was approved by the Ethics Committee of Wuhan Wudong Hospital (approval number: WDYY-LL-2021-12, Date: December 30, 2021).

The inclusion criteria were as follows: (1) patients who met the diagnostic criteria for manic episodes outlined in the Chinese Classification and Diagnostic Criteria for Mental Disorders, 3rd Edition [12], (2) patients with a total Bech–Rafaelsen Manic Rating Scale score ≥6 points with no obvious impulsive behavior, and (3) patients aged ≥18 years.

The exclusion criteria were as follows: (1) patients who had used psychiatric drugs 2 weeks prior to admission, (2) patients who used long-acting injections, a combination of multiple mood stabilizers or antipsychotics, or antidepressants, (3) patients with acute or chronic nephritis, severe cardiovascular disease, acute infection, organic diseases of the central nervous system, and/or clear renal insufficiency.

Sample size was determined using G*Power 3.1 software (Heinrich-Heine-University Düsseldorf, Düsseldorf, Germany), assuming a medium effect size (*f* = 0.25), using an α-value of 0.05 and a power of 0.80 with four groups for analysis of variance, which resulted in a required sample size of 180. The total sample size of 644 patients in this study far exceeds this requirement.

The Bech-Rafaelsen Mania Rating Scale (BRMS) [3] is a clinical tool used to assess the severity of manic symptoms. It is widely applied in the diagnosis and evaluation of treatment efficacy for bipolar disorder (manic-depressive illness) and other psychiatric disorders. Below is detailed information about this scale:

Scale Structure: The BRMS consists of 11 core items, which assess manic symptoms in various aspects of patients, including mood, speech, flight of ideas, activity level, sleep disturbance, irritability, and increased sexual interest. In addition, the scale collaboration group has added 2 supplementary items (hallucinations and delusions), bringing the total number of items to 13.

Scoring Method: Scoring System: A five-point rating scale is used. Each item is scored based on the severity of the symptom, ranging from 0 (no symptoms) to 4 (extremely severe symptoms). Scoring Basis: The rating is completed by evaluators through interviews with patients, observations, and inquiries to family members or ward staff. Time Frame: The initial rating is usually based on the patient’s performance over the past week, and subsequent ratings are generally conducted within 2 to 6 weeks.

Total Score Calculation: The total score is obtained by summing up the scores of all items, with a range of 0 to 44 points.

Interpretation of Total Score: 0–5 points: No significant manic symptoms. 6–10 points: Mild manic symptoms. 11–14 points: Hypomania. 15–24 points: Moderate mania. 25–44 points: Severe mania. The higher the total score, the more severe the manic symptoms. Changes in the total score before and after treatment can reflect therapeutic efficacy, with a larger difference indicating better therapeutic outcomes.

Assessors: Typically, the assessment is conducted by clinically trained doctors or psychiatric nurses. Professional Requirements: Assessors need to have certain clinical experience in psychiatry and training in scale usage to ensure the accuracy and consistency of scoring. Reliability: Statistical data from the domestic scale collaboration group show that the BRMS has high inter-rater reliability (r = 0.97–0.99), indicating good reliability. Validity: The total score of the BRMS has a strong correlation with the clinical judgment of the severity of mania (r = 0.92), suggesting high validity.

### 2.2 Lithium Carbonate Therapy

Patients were given oral lithium carbonate (H10900013, Enhua, Xuzhou, Jiangsu, China) at an initial dose of 0.9–1.5 g per day for 1 week that was reduced to a maintenance dose of 0.6–0.9 g per day thereafter. The specific dosage within this range for each patient was determined by their treating psychiatrist based on individual clinical factors. The drug was administered immediately after meals and at dinner (at 17:00). Patients were not allowed any other mood stabilizers, antipsychotics, angiotensin-converting enzyme inhibitors, non-steroidal anti-inflammatory drugs, diuretics, phenytoin, or certain antibiotics during the study period.

All enrolled patients received a common diet provided by the hospital, with a total energy intake of about 10,460 kJ/day. The target energy intake of 10,460 kJ/day was selected based on the findings of a systematic review by McKenzie *et al*. (2022) [13]. While we aimed for all patients to meet this intake, individual variations in appetite and food consumption occurred.

### 2.3 Detection of Drug Concentration and Other Biochemical Indicators

Serum lithium concentration was measured 7 days after initiation of the maintenance dose. Two mL venous blood was drawn 12 hours after the last dose, to represent the 12-hour standard serum lithium concentration [14]. Serum lithium concentrations were measured using the electrode method (Caretium XI-921ET, Shenzhen, Guangdong, China).

Concentration/dose ratio values were calculated as follows: steady-state lithium plasma concentration/oral dose.

Uric acid, blood glucose (GLU), and triglyceride (TG) levels were measured using an automatic biochemical analyzer (Mindray BS820, Shenzhen, Guangdong, China).

### 2.4 Grouping Methods

Blood pressure (BP) was measured daily during hospitalization. Patients were considered to have hypertension if they had a pre-existing diagnosis of hypertension or if they consistently showed an elevated BP (systolic BP ≥130 mmHg or BP diastolic ≥80 mmHg) for at least 3 consecutive days during their hospital stay. Patients were divided into groups based on the following criteria: a systolic BP ≥130 mmHg was considered high; a systolic BP <130 mmHg was considered non-high; a diastolic BP ≥80 mmHg was considered high; a diastolic BP <80 mmHg was considered non-high; patients with serum UA ≥420 µmol/L were considered hyperuricemic; patients with a serum UA <420 µmol/L were considered non-hyperuricemic; patients with a serum GLU ≥6.11 mmol/L were considered hyperglycemic; patients with a serum GLU <6.11 mmol/L were considered non-hyperglycemic; a serum TG ≥2.3 mmol/L was considered high; and a serum TG <2.3 mmol/L was considered non-high. Blood glucose, TG, and UA levels were measured upon admission and regularly throughout the hospitalization period as part of standard clinical care.

### 2.5 Statistical Methods

Analysis was completed using SPSS v. 17.0 statistical software (SPSS Inc., Chicago, IL, USA). A normality test was performed using the Kolmogorov–Smirnov method. Measurement data describing normality were expressed as mean ± standard deviation ($\bar{x}$ ± *s*). Independent sample *t*-tests were used for comparisons between groups. A Pearson’s correlation analysis was used to assess relationships between variables.

In addition to univariate analyses, the team performed multiple linear regressions to assess the relationship between C/D value and the study’s variables of interest (systolic BP, diastolic BP, GLU, TGs, and UA) while controlling for potential confounders, such as age, sex, and body mass index (BMI).

Statistical significance was established at *p* < 0.05.

## 3. Results

### 3.1 General Information

Of the 644 patients included in this study, 321 were men and 323 were women. The mean age was 30.70 ± 8.57 years, the mean BMI was 22.37 ± 3.21 kg/m^2^, and the mean course of disease was 21.46 ± 7.32 years. The mean Bech-Rafaelsen Mania Rating Scale score at admission was 22.3 ± 6.5, indicating moderate to severe manic symptoms. Detailed demographic and clinical characteristics are shown in Table 1.

**Table 1.** **General information of study participants**.

Characteristic		Value	Proportion (%)
Gender			
	Male	321	49.84
	Female	323	50.16
Age (years)		30.70 ± 8.57	-
BMI (kg/m^2^)		22.37 ± 3.21	-
BRMS score at admission		22.3 ± 6.5	-
Course of disease (years)		21.46 ± 7.32	-
Family history			
	Yes	154	23.91
	No	490	76.09
Diabetes			
	Yes	87	13.51
	No	557	86.49
Initial dose of oral lithium carbonate (g/d)		1.21 ± 0.25	-
Systolic blood pressure (mmHg)		128.5 ± 15.3	-
Diastolic blood pressure (mmHg)		79.2 ± 9.7	-
Blood glucose (mmol/L)		5.4 ± 1.2	-
Triglycerides (mmol/L)		1.8 ± 0.9	-
Uric acid (µmol/L)		385.6 ± 98.4	-

BMI, body mass index; BRMS, Bech-Rafaelsen Mania Rating Scale.

### 3.2 Comparison of Serum Lithium and C/D Values in Different Groups

The mean blood lithium C/D value for all patients in this study was 0.832 ± 0.248 mmol⋅L^-1^⋅g^-1^⋅d. Comparisons of serum lithium concentrations and C/D values between different groups are shown in Table 2. Notably, C/D values in the high systolic BP group (0.868 ± 0.265) were significantly lower than those in the non-high systolic BP group (0.954 ± 0.337; *t* = –2.845, *p* = 0.007). Concentration/dose ratio values in the hyperuricemic group (0.923 ± 0.279) were also significantly lower than those in the non-hyperuricemic group (0.939 ± 0.350; *t* = 0.615, *p* = 0.000).

**Table 2.** **Comparison of serum lithium and C/D values in different groups**.

Grouping indicator	Group	Serum lithium concentration	C/D value
Systolic blood pressure	NHSBP (n = 488)	0.592 ± 0.207	0.954 ± 0.337
	HSBP (n = 156)	0.551 ± 0.154	0.868 ± 0.265
	t/*p* values	2.28/0.0229	–2.845/0.007
Diastolic blood pressure	NHDBP (n = 382)	0.601 ± 0.215	0.977 ± 0.348
	HDBP (n = 262)	0.556 ± 0.161	0.868 ± 0.271
	t/*p* values	0.015/0.988	0.720/0.458
Blood glucose	NHGLU (n = 567)	0.582 ± 0.197	0.930 ± 0.326
	HGLU (n = 77)	0.577 ± 0.185	0.947 ± 0.299
	t/*p* values	0.221/0.833	–0.542/0.591
Triglyceride	NHTG (n = 542)	0.572 ± 0.168	0.934 ± 0.317
	HTG (n = 102)	0.631 ± 0.293	0.924 ± 0.352
	t/*p* values	–1.967/0.058	0.517/0.608
Uric acid	NHUA (n = 386)	0.556 ± 0.208	0.939 ± 0.350
	HUA (n = 258)	0.617 ± 0.174	0.923 ± 0.279
	t/*p* values	–3.888/0.001	0.615/0.000

C/D, concentration/dose ratio; NHSBP, Non High Systolic blood pressure; HSBP, High Systolic blood pressure; NHDBP, Non High Diastolic blood pressure; HDBP, High Diastolic blood pressure; NHGLU, Non High Blood glucose; HGLU, High Blood glucose; NHTG, Non High Triglyceride; HTG, High Triglyceride; NHUA, No High Uric acid; HUA, High Uric acid.

### 3.3 Correlation Analysis

Correlation analysis results between different physiological indicators, serum lithium concentrations, and C/D values are presented in Table 3. Systolic BPs were negatively correlated with C/D values (*r* = –0.232; *p* = 0.001), as were UA levels (*r* = –0.114; *p* = 0.013). No significant correlations were found for any other indicators.

**Table 3.** **Correlation analysis between physiological indicators and serum lithium and C/D values**.

Grouping indicator	Serum lithium concentration		C/D value	
	*r*	*p*	*r*	*p*
Systolic blood pressure	0.123	0.231	–0.232	0.001
Diastolic blood pressure	0.114	0.341	0.113	0.142
Blood glucose	0.078	0.112	–0.110	0.193
Triglyceride	–0.021	0.322	0.094	0.092
Uric acid	–0.124	0.098	–0.114	0.013

### 3.4 Multiple Linear Regression Analysis

To control for potential confounding factors, we performed a multiple linear regression analysis with C/D value as the dependent variable and systolic BP; diastolic BP; GLU, TG, and UA levels; age; sex; and BMI as independent variables. The results confirmed that systolic BP (β = –0.215; *p* = 0.003) and UA (β = –0.109; *p* = 0.018) were independently associated with C/D value, even after adjusting for other factors. β represents the standardized regression coefficient.

### 3.5 Safety Monitoring

Throughout the study, patients were monitored for signs of lithium toxicity. While some patients experienced mild side effects, such as nausea or tremors, no cases of severe lithium toxicity were observed.

## 4. Discussion

This study investigated the effects of various physiological factors, particularly BP and UA level, on blood lithium C/D values in patients with manic episodes. Our findings reveal that hypertension and hyperuricemia each have significant associations with lithium pharmacokinetics. This revelation has important implications for the clinical management of patients receiving lithium therapy.

The mean blood lithium C/D value observed in our study (0.832 ± 0.248 mmol⋅L^-1^⋅g^-1^⋅d) is consistent with previous research, suggesting that our patient population is representative of typical lithium-treated individuals with manic episodes [15]. However, we found significant variations in C/D values associated with certain physiological factors—specifically systolic BP and Uric acid levels.

Our results show that patients with abnormal systolic BPs (>130 mmHg) had significantly lower lithium C/D values compared with those with normal systolic BPs. This finding was further supported by the negative correlation between systolic BP and C/D value (*r* = –0.232; *p* = 0.001). This relationship persisted even after controlling for potential confounding factors in our multiple regression analysis.

The inverse relationship between systolic BP and lithium C/D value is an intriguing finding that warrants further investigation. One possible explanation is that hypertension may affect renal blood flow and GFR, which are crucial factors in lithium clearance. Previous studies have shown that hypertension can lead to changes in renal structure and function, potentially altering drug pharmacokinetics [16].

Similarly, we found a negative correlation between UA levels and C/D values (*r* = –0.114, *p* = 0.013), with patients in the hyperuricemic group showing lower C/D values than those in the non-hyperuricemic group. This relationship also remained significant in our multiple regression analysis, suggesting an independent effect of UA level on lithium pharmacokinetics.

The association between UA level and lithium C/D value is a novel finding that has not been extensively explored in previous studies. Elevated UA levels are often associated with metabolic disorders and may reflect underlying changes in renal function [17]. It is possible that the mechanisms influencing UA level also affect lithium metabolism—or that UA itself has a direct effect on lithium pharmacokinetics.

These findings have important clinical implications. The lower C/D values observed in patients with high systolic BP and elevated UA levels suggest that these individuals may require higher lithium doses to achieve therapeutic serum concentrations. Conversely, if hypertension or hyperuricemia develop during lithium treatment, dose reductions may be necessary to prevent toxicity.

Our results underscore the importance of regular BP and UA level monitoring in patients receiving lithium therapy. Changes in these parameters may necessitate adjustments in lithium dosing to maintain therapeutic efficacy while minimizing the risk of toxicity. This aligns with the growing emphasis on personalized medicine in psychiatry, wherein treatment is tailored to individual patient characteristics [18].

It is worth noting that we did not find significant associations between lithium C/D value and other factors, such as diastolic BP, and GLU and TG levels. This suggests that these factors may have less influence on lithium pharmacokinetics, although further research with larger sample sizes may be needed to confirm these findings.

### 4.1 Limitations

This study has several limitations that should be considered. First, as an observational study, we cannot establish causal relationships between the observed physiological factors and lithium C/D value. Second, while we controlled for several potential confounding factors, there may be other unmeasured variables influencing our results. Third, our study was conducted at a single center, which may limit its generalizability to other populations.

### 4.2 Future Directions

Future research should focus on elucidating the mechanisms underlying the observed relationships between systolic BP, UA level, and lithium C/D value. Prospective studies examining how changes in these physiological factors over time affect lithium pharmacokinetics would be particularly valuable. Additionally, clinical trials investigating whether personalized dosing strategies based on these factors improve treatment outcomes would be an important next step in translating these findings into clinical practice.

## 5. Conclusion

Our study demonstrates that hypertension and elevated Uric acid level are associated with lower blood lithium C/D values in patients with manic episodes. These findings suggest the need for personalized lithium dosing strategies that take account of these physiological factors. Regular monitoring of BP and UA level may help to optimize lithium treatment, potentially improving efficacy and reducing the risk of toxicity. Further research is needed to fully understand the mechanisms underlying these relationships and to develop evidence-based guidelines for personalized lithium therapy in patients with manic episodes.

## Availability of Data and Materials

The datasets used and/or analyzed during the current study are available from the corresponding authors on reasonable request.

## References

[1] Goodwin GM (2020). Bipolar disorder. *Medicine*.

[2] Yatham LN, Chakrabarty T, Bond DJ, Schaffer A, Beaulieu S, Parikh SV (2021). Canadian Network for Mood and Anxiety Treatments (CANMAT) and International Society for Bipolar Disorders (ISBD) recommendations for the management of patients with bipolar disorder with mixed presentations. *Bipolar Disorders*.

[3] Delvecchio G, Mandolini GM, Perlini C, Barillari M, Marinelli V, Ruggeri M (2018). Pituitary gland shrinkage in bipolar disorder: The role of gender. *Comprehensive Psychiatry*.

[4] Malhi GS, Bell E, Outhred T, Berk M (2020). Lithium therapy and its interactions. *Australian Prescriber*.

[5] Sinha A, Shariq A, Said K, Sharma A, Jeffrey Newport D, Salloum IM (2018). Medical Comorbidities in Bipolar Disorder. *Current Psychiatry Reports*.

[6] Oparil S, Acelajado MC, Bakris GL, Berlowitz DR, Cífková R, Dominiczak AF (2018). Hypertension. *Nature Reviews. Disease Primers*.

[7] Levey AS, Eckardt KU, Tsukamoto Y, Levin A, Coresh J, Rossert J (2005). Definition and classification of chronic kidney disease: a position statement from Kidney Disease: Improving Global Outcomes (KDIGO). *Kidney International*.

[8] Gitlin M (2016). Lithium side effects and toxicity: prevalence and management strategies. *International Journal of Bipolar Disorders*.

[9] Severus WE, Kleindienst N, Seemüller F, Frangou S, Möller HJ, Greil W (2008). What is the optimal serum lithium level in the long-term treatment of bipolar disorder-a review?. *Bipolar Disorders*.

[10] McKnight RF, Adida M, Budge K, Stockton S, Goodwin GM, Geddes JR (2012). Lithium toxicity profile: a systematic review and meta-analysis. *Lancet (London, England)*.

[11] Lazarus JH (2009). Lithium and thyroid. Best Practice & Research. *Clinical Endocrinology & Metabolism*.

[12] Chen YF (2002). Chinese classification of mental disorders (CCMD-3): towards integration in international classification. *Psychopathology*.

[13] McKenzie YA, Sremanakova J, Todd C, Burden S (2022). Effectiveness of diet, psychological, and exercise therapies for the management of bile acid diarrhoea in adults: A systematic review. *Journal of Human Nutrition and Dietetics: the Official Journal of the British Dietetic Association*.

[14] Hiemke C, Baumann P, Bergemann N, Conca A, Dietmaier O, Egberts K (2011). AGNP Consensus Guidelines for Therapeutic Drug Monitoring in Psychiatry: Update 2011. *Pharmacopsychiatry*.

[15] Hiemke C, Bergemann N, Clement HW, Conca A, Deckert J, Domschke K (2018). Consensus Guidelines for Therapeutic Drug Monitoring in Neuropsychopharmacology: Update 2017. *Pharmacopsychiatry*.

[16] Webster AC, Nagler EV, Morton RL, Masson P (2017). Chronic Kidney Disease. *Lancet (London, England)*.

[17] Borghi C, Rosei EA, Bardin T, Dawson J, Dominiczak A, Kielstein JT (2015). Serum uric acid and the risk of cardiovascular and renal disease. *Journal of Hypertension*.

[18] Salazar de Pablo G, Studerus E, Vaquerizo-Serrano J, Irving J, Catalan A, Oliver D (2021). Implementing Precision Psychiatry: A Systematic Review of Individualized Prediction Models for Clinical Practice. *Schizophrenia Bulletin*.

